# Eye Injuries in Film Production

**Published:** 1921-09-24

**Authors:** 


### Eye Injuries in Film Production.

An Interim Report of the Departmental Committee on the Causes and Prevention of Blindness has been issued by H.M. Stationery Office. Price 3d. net. The Committee was appointed by the Minister of Health to investigate the causes and prevention of blindness, with the Rt. Hon. G. H. Roberts as Chairman. The Report deals with the injuries to the sight of actors and actresses alleged to be due to the powerful lights used for the production of films in kinematograph studios concerning which complaints were made by the Actors' Association last year. The Committee trace the trouble to the use of open arc lights without diffusing screens. They find that some transient eye injuries have thus been caused, but that there is no evidence of permanent damage to sight. They state that the evidence given them is to the effect that unscreened arcs are not only unnecessary, but give less satisfactory photographic results, and the Incorporated Association of Kinematograph Manufacturers have given an undertaking to the Minister of Health that its members will not permit the use of open-arc lights without filters in their studios. The Committee accept this undertaking, but point out that the industry is in a state of development, and that research is needed as to the best types of lamp required.

